# Cobalt Ferrite Particles Produced by Sol-Gel Autocombustion and Embedded in Polysilane: An Innovative Route to Magnetically-Induced Fluorescence Composites

**DOI:** 10.3390/molecules27196393

**Published:** 2022-09-27

**Authors:** Petrisor Samoila, Corneliu Cojocaru, Mihaela Simionescu, Gabriela Sacarescu, Gheorghe Roman, Andra-Cristina Enache, Liviu Sacarescu

**Affiliations:** Laboratory of Inorganic Polymers, “Petru Poni” Institute of Macromolecular Chemistry, 41A Grigore Ghica Voda Alley, 700487 Iasi, Romania

**Keywords:** fluorescence, polysilane, cobalt ferrite, sol-gel autocombustion, composite, core-shell

## Abstract

Fluorescence detection is currently one of the commonly used techniques worldwide. Through this work, the preparation and optical properties of an interesting composite material are discussed. It is shown that encapsulating cobalt spinel ferrite (CoFe_2_O_4_), obtained by the sol-gel autocombustion method, into poly[diphenyl-co-methyl(H)]silane matrix leads to fluoromagnetic particles (PSCo) with intriguing optical properties. Transmission electron microscopy, combined with energy-dispersive X-ray analysis, showed 500 nm large spherical structures containing a core (around 400 nm in diameter) composed of magnetic ferrite particles, surrounded by a thin layer of semiconductive fluorescent polymer. The as-obtained material exhibited ferrimagnetic properties. The FTIR spectrum confirmed that the Si-H functionality of the polysilane was preserved. UV spectroscopy combined with molecular modeling studies indicated that the magnetic core had a strong influence on the intramolecular electron transitions characteristic of the σ-conjugated polysilane. Further analysis by steady-state fluorescence spectroscopy revealed that the internal magnetic field strongly enhances the polysilane emission. This property will be further investigated in the future in order to develop new detection devices.

## 1. Introduction

Fluorescent materials are recognized as an attractive option in the field of detection due to the versatility of preparation methods and the diversity of applications. For this reason, the interest in research and exploration of various techniques aiming to obtain such systems has grown exponentially [1,2,3,4,5,6,7]. Currently, there are a large number of papers presenting various kinds of compounds with fluorescent properties that respond under the action of light stimuli [8,9,10]. Among these, fluorescent polymers have received particular attention [11,12]. The main advantage of these materials consists in their easy processing in the form of films, particles, gels, or capsules, a fact that further widens their application area [13,14,15,16].

From this point of view, there is an interesting class of macromolecular compounds generically called polysilanes [17]. These σ–π conjugated polymers are characterized by a particular chemical structure in which variously substituted silicon atoms are directly connected to form long chains. Such a structure, intensively studied and presented in the literature, refers to polydiphenylsilane [18]. This polymer stands out through its optical and electronic properties, which make it an excellent candidate as an intrinsic semiconductor in microelectronics applications [19]. Nevertheless, a major disadvantage of this compound is its insolubility, imposing difficult processing conditions [20]. However, a number of studies have shown that including short segments of methylhydrosilyl in the polydiphenylsilane chain leads to enhanced solubility in common organic solvents, while the optoelectronic properties, semiconductivity and fluorescence, are not affected [21,22]. In addition, the Si–H groups allow for derivation and building unconventional architectures with interesting properties, which are useful in various applications [23,24,25]. Moreover, polysilanes are biocompatible, making them suitable for the biomedical field [26].

One of the most common approaches, when the application has a high degree of complexity or a very specific one [27,28,29,30], is to create a composite system that, in addition to the fluorescent component, contains one or more of the other constituents with different properties that combine synergistically.

In this context, magnetic (nano)particles (MNPs) represent a very attractive category of materials that can induce new properties to their composites, such as magnetic susceptibility and the possibility of manipulation under an external field [31,32,33]. Usually, MNPs perform the role of transport vectors and their response capacity under the action of magnetic field is influenced by their magnetic properties, in accordance with their chemical composition and structure (pure metallic MNPs, metal oxide MNPs, and alloy MNPs) [34,35,36].

An interesting category of MNPs is that of ferrites with a spinel structure. These mixed oxide materials have the general formula MFe_2_O_3_ (where M is a divalent cation such as Fe, Zn, Ni, or Co). Spinel ferrites have optical and magnetic properties that can be adjusted by their chemical composition, depending on the targeted applications, either for microelectronics or as (photo)catalysts [37,38]. The latter property has gained special significance in the context of wastewater depollution activity. One of the most representative compounds of spinel ferrite class is cobalt ferrite (CoFe_2_O_4_). Cobalt ferrite is a semiconductor oxide that is characterized by cubic magnetocrystalline anisotropy. This material has high chemical stability, low toxicity, moderate magnetization, and high coercivity [39,40,41,42]. There are a number of papers that describe the synthesis methods as well as the properties of this material [37]. Most of the works present the wet chemical methods, in general, and sol-gel method, in particular, as the most suitable approaches for spinel ferrites synthesis because of its advantages such as very good homogeneity, narrow grain size distribution, phase purity, and low-cost [37,38].

The magnetic field generated by MNPs can strongly influence the properties of composite materials. Thus, in some cases, the magnetic field exerted by MNPs can lead to a substantial amplification of the composite fluorescence [43,44,45]. This effect underlies one of the most versatile methods of regulating emission properties for a wide range of composite systems, including quantum dots and organic semiconductors. Known as magnetic fluorescence enhancement (MFE), the phenomenon has been reported and described especially for hard materials, with an external magnetic field applied [46,47,48,49]. However, such a system is unseemly in terms of its ability to adjust the material structure and the equipment needed to apply the external magnetic field. One way to overcome these obstacles is based on doping the fluorescent component with magnetic particles to obtain MFE.

This paper presents a particular case of MFE for a composite material obtained by mixing a fluorescent polymer, polydiphenyl(H)silane (PSH), with cobalt ferrite, CoFe_2_O_4_, prepared by sol-gel autocombustion method. UV irradiation of the fluorescent component produces charge carriers that interact electromagnetically with ferrite particles, generating a weak magnetic field and thus influencing the emission intensity.

## 2. Results and Discussion

### 2.1. Transmission Electron Microscopy (TEM)

**Figure 1:**
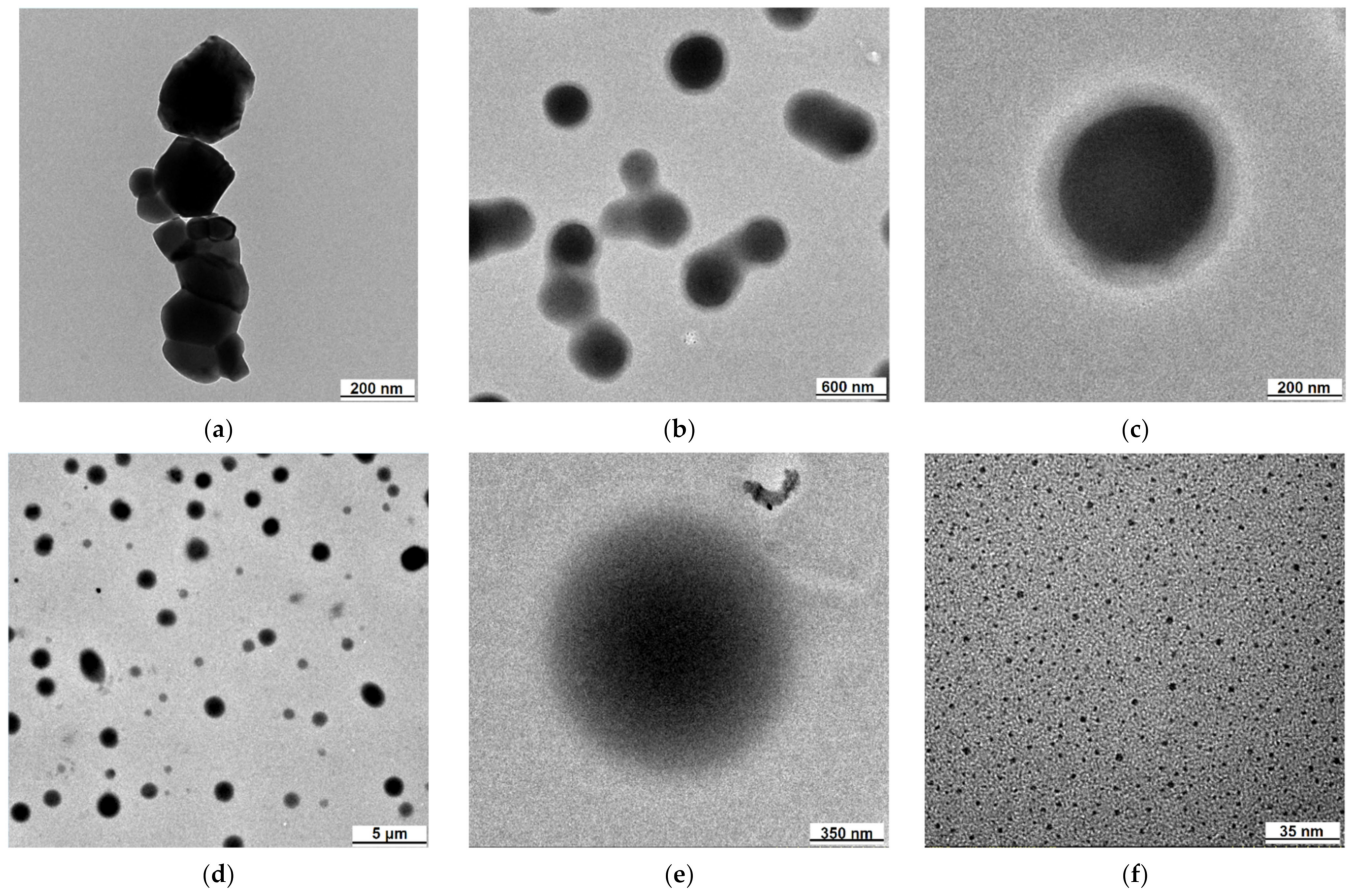
Representative transmission electron microscopy images for (**a**) Co spinel ferrite; (**b**) polysilane-Co ferrite particles (PSCo); (**c**) close-up of a single PSCo fluoromagnetic structure; (**d**) polysilane (PSH) dispersion in methanol; (**e**) close-up of PSH dispersion in methanol; and (**f**) Co ferrite particles after ultrasound processing and washing in tetrahydrofuran (THF).

**Figure 2:**
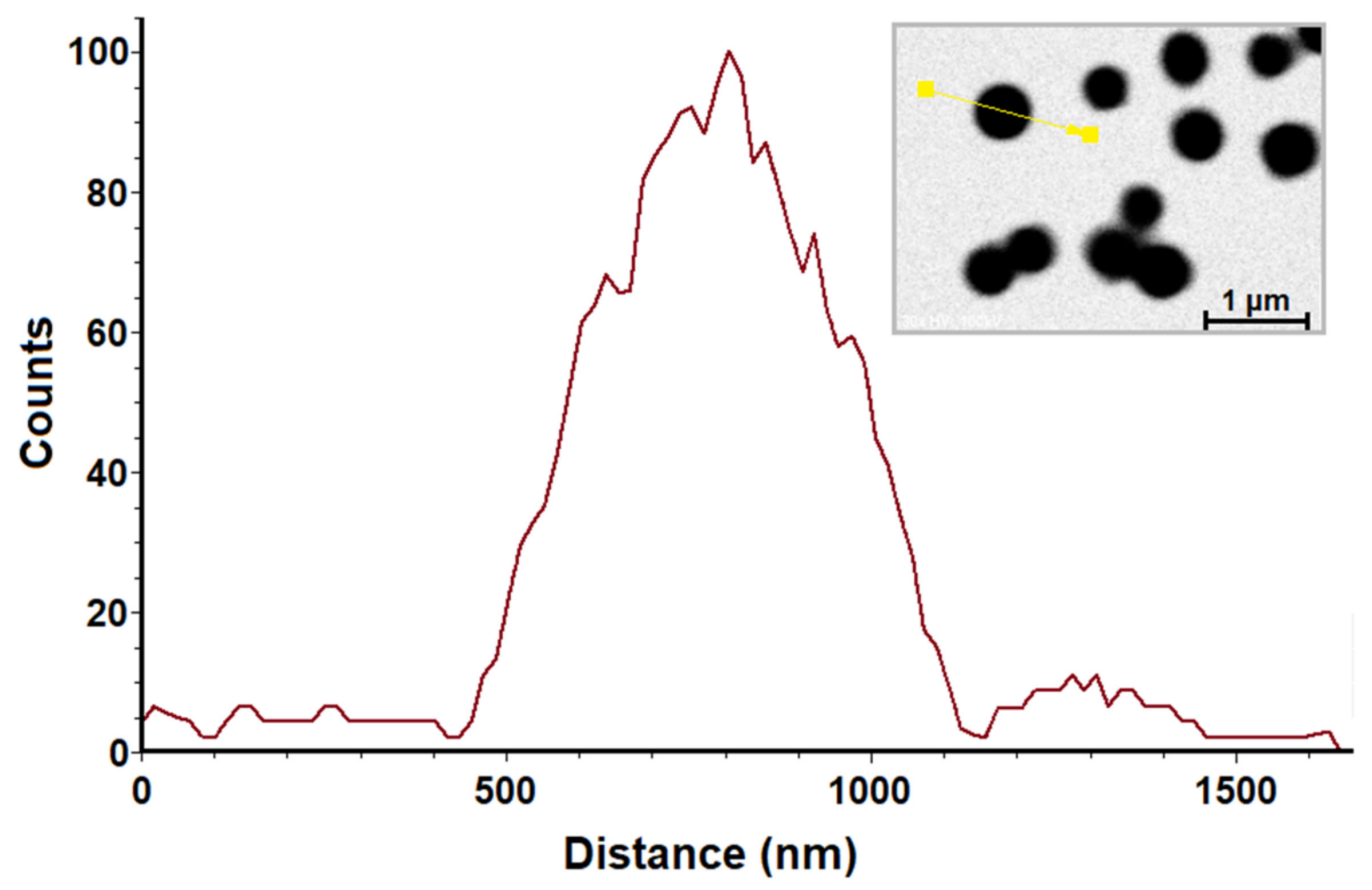
Energy dispersive X-ray (EDX) line profile showing a higher oxygen content within a PSCo particle.

The specific morphology of each category of (nano)particles was described using transmission electron microscopy (TEM). It was thus observed that the polysilane-cobalt ferrite (PSCo) fluoromagnetic particles are shaped as spherical structures of relatively large dimensions, with an average diameter of about 500 nm (Figure 1b,c). These contain a dense core surrounded by a layer-shell of approximately 40–60 nm that is evenly distributed. In order to obtain information on the constituent elements of the particles and especially of the dense core, an energy-dispersive X-ray analysis (EDX) was performed. The EDX line profile analysis confirmed that CoFe_2_O_4_ is concentrated in this core, due to the much higher oxygen concentration inside the structure (Figure 2). Therefore, fluoromagnetic particles contain a dense magnetic core, around which there is the polysilane film.

Although initially the ferrite particles have irregular shapes, a very wide dimensional polydispersity, and an average size of 201 nm [50] (Figure 1a), the core of the fluoromagnetic particles are perfectly spherical. Additionally, TEM analysis of PSH dispersions in methanol indicates the presence of very large (in the micron domain) spherical polymer particles (Figure 1d,e). In the case of fluoromagnetic structures, the presence of the polymer is only found in the film that envelops the magnetic core. On the other hand, TEM analysis of the ferrite particles extracted from the final PSCo composite by washing in tetrahydrofuran (THF) shows very small dimensions (Figure 1f).

One can thus imagine the mechanism by which the PSCo fluoromagnetic particles were formed. The ultrasonic step, aiming to disperse the ferrite particles in a polymer solution, might also lead to the crushing of some grains to nanometric dimensions (<10 nm). Another hypothesis is that, by utrasonication, the nanometric grains of CoFe_2_O_4_ attached to the micrometric ones due to their magnetic properties and were dispersed in the polymeric solution. Subsequently, by precipitation in methanol, globular polymer structures are formed, structures that incorporate a significant number of very small ferrite particles in the form of a dense core, most probably due to their magnetic properties. Thus, the final structure of the magneto-fluorescent particles acquires the appearance of a core-shell.

### 2.2. Fourier-Transform Infrared Spectroscopy (FTIR)

**Figure 3:**
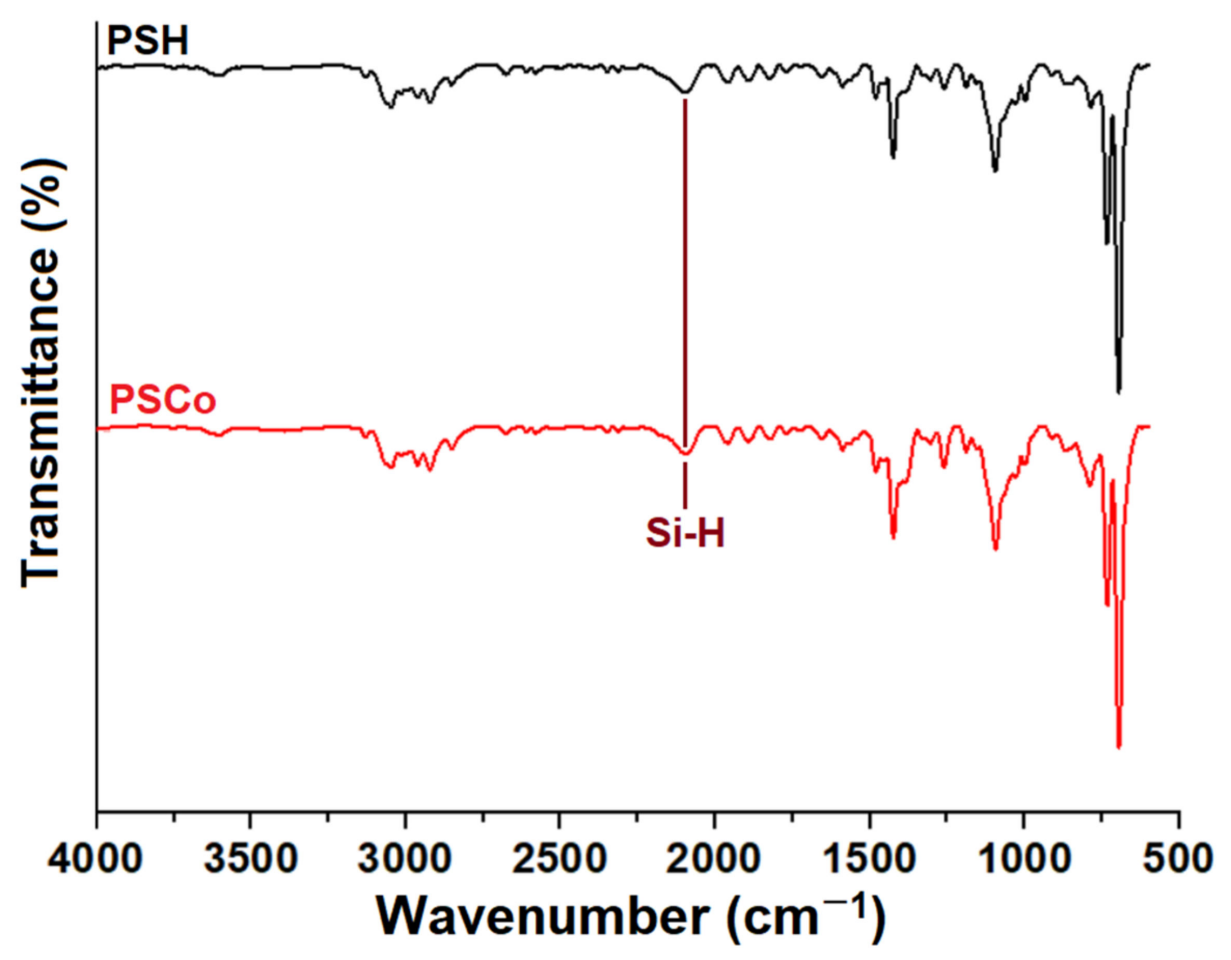
FTIR spectra of PSH and PSCo showing the presence of the Si–H functionality in the fluoromagnetic particles.

FTIR analysis confirms the presence of PSH in the structure of PSCo particles (Figure 3). One can note the presence of a particular IR shift corresponding to the Si–H bond (2907 cm^−1^). This confirms that the chemical structure of polysilane remains unchanged and retains its functionality. This fact is important because the Si-H group can be further used in various chemical processes to change the polymer chemical structure by attaching molecular segments, which can provide additional properties to the fluoromagnetic particles.

### 2.3. Magnetic Properties

**Figure 4:**
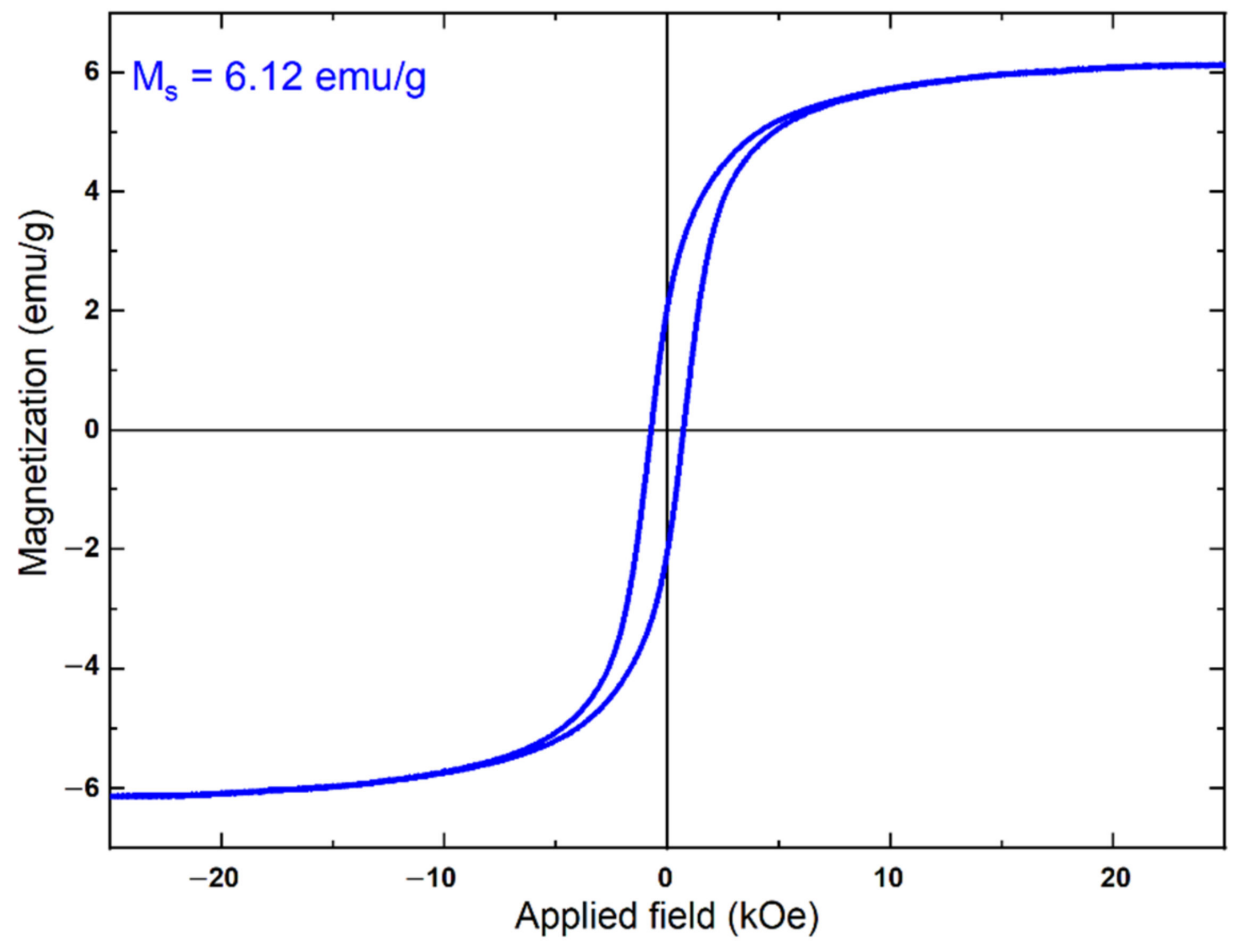
Room temperature hysteresis loop for PSCo sample.

The hysteresis loop registered at room temperature and the saturation magnetization value are displayed in Figure 4. The PSCo loop is typical for a ferrimagnetic material and indicates that the magnetization of the sample reached saturation when 10 kOe external magnetic field was applied. The saturation magnetization (Ms) for the composite is equal to 6.12 emu/g. For comparison, the cobalt ferrite (CoFe_2_O_4_) has a Ms value of 67.22 emu/g [50]. Hence, the relative ratio of magnetic saturation for the composite and the pure cobalt ferrite is about 9.10%. This result is consistent since, in the composite, the weight fraction of ferrite is equal to 8.33%. This means that the presence of the polymer does not influence the behavior of the magnetic component.

### 2.4. Optical Properties

**Figure 5:**
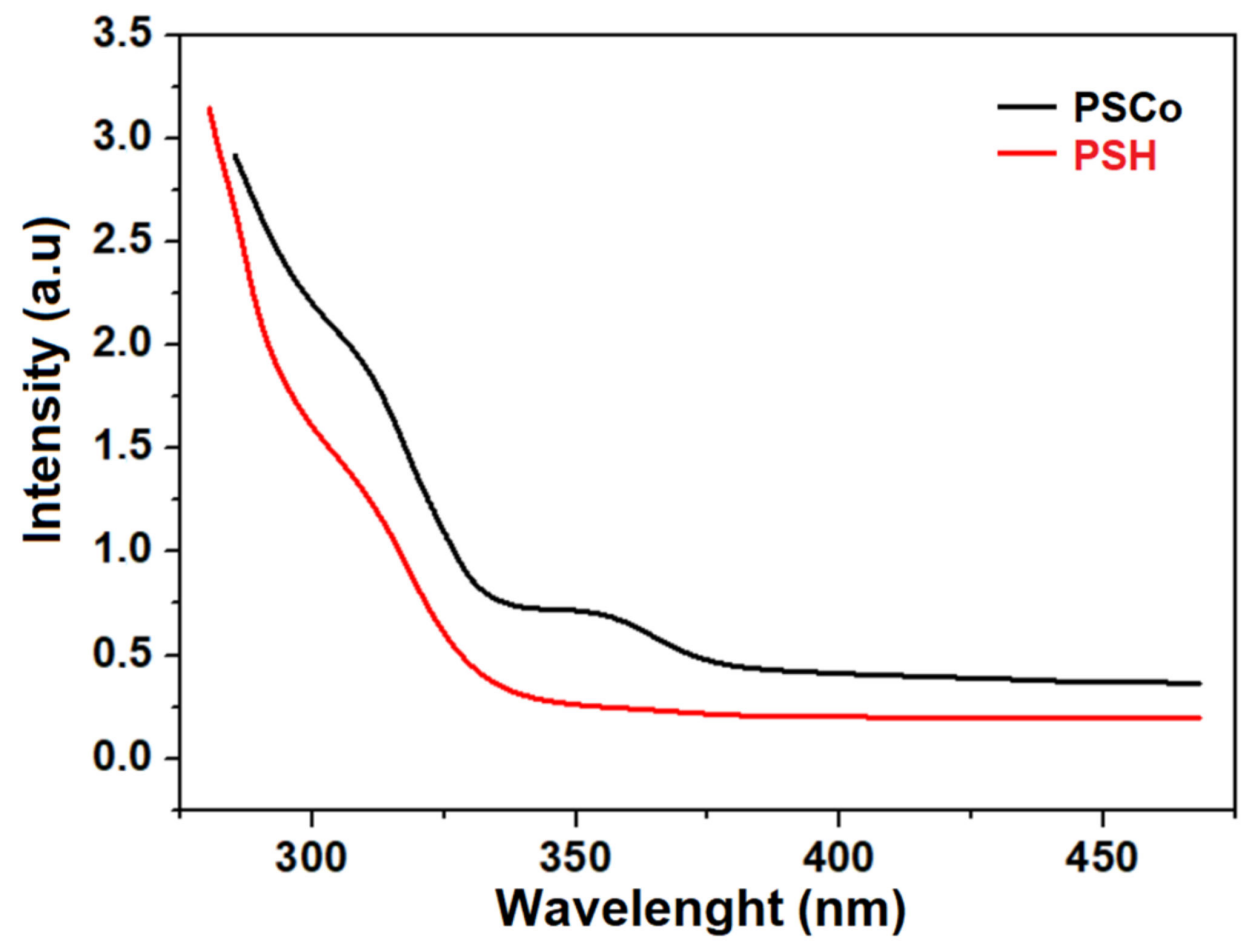
The UV absorption spectra of PSCo and PSH samples.

Usually, the UV absorption spectrum of PSH solution displays a characteristic profile with two main absorption bands [22]. The one located at around 350 nm originates from the σ → σ* UV electron transition, while the second one, at around 280 nm, was assigned to the π → π* electron transitions in the aromatic ring. On the other hand, the UV absorption spectra of PSH dispersion in methanol has a different profile, without showing the characteristic 350 nm absorption band (Figure 5). By contrast, the UV absorption profile of PSCo dispersion shows the presence of this specific electron transition (Figure 5). Therefore, in the case of PSH, the presence of methanol molecules strongly attenuates the 350 nm UV absorption band, which does not occur in the case of PSCo.

**Scheme 1:**
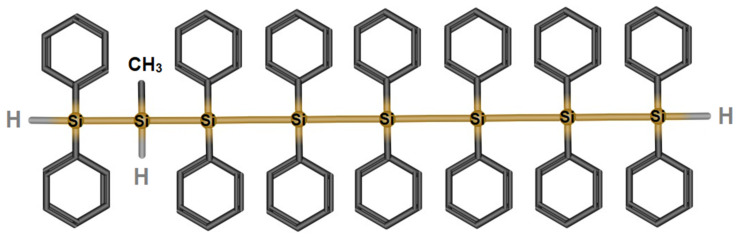
Chemical formula (2D geometry) of the investigated oligomer PSH.

In order to understand the origin of this different optical behavior, it is necessary to verify first if the presence of the methanol molecules around the PSH chain could attenuate the UV absorption band at 350 nm. Details concerning this aspect were obtained using computational chemistry. The modeled polysilane PSH taken into account for this purpose is an oligomeric structure with eight Si-atoms in the backbone chain, 14 phenyl groups, one methyl moiety, and three hydrogen atoms (Scheme 1).

First, the molecular conformation of PSH was optimized (in gas phase) by the PM6 method (semi-empirical), and then its electronic structure was explored in more advanced fashion by using the density functional theory (DFT) and time-dependent density functional theory (TD-DFT) methods at the B3LYP/6-311G** level of theory [51]. Second, the influence of methanol (explicit solvent molecules) on the theoretical UV-Vis spectrum of PSH was also investigated. In this respect, the PSH oligomer surrounded by 14 molecules of CH_3_OH was also subjected to TD-DFT calculations to account for the excited states.

**Figure 6:**
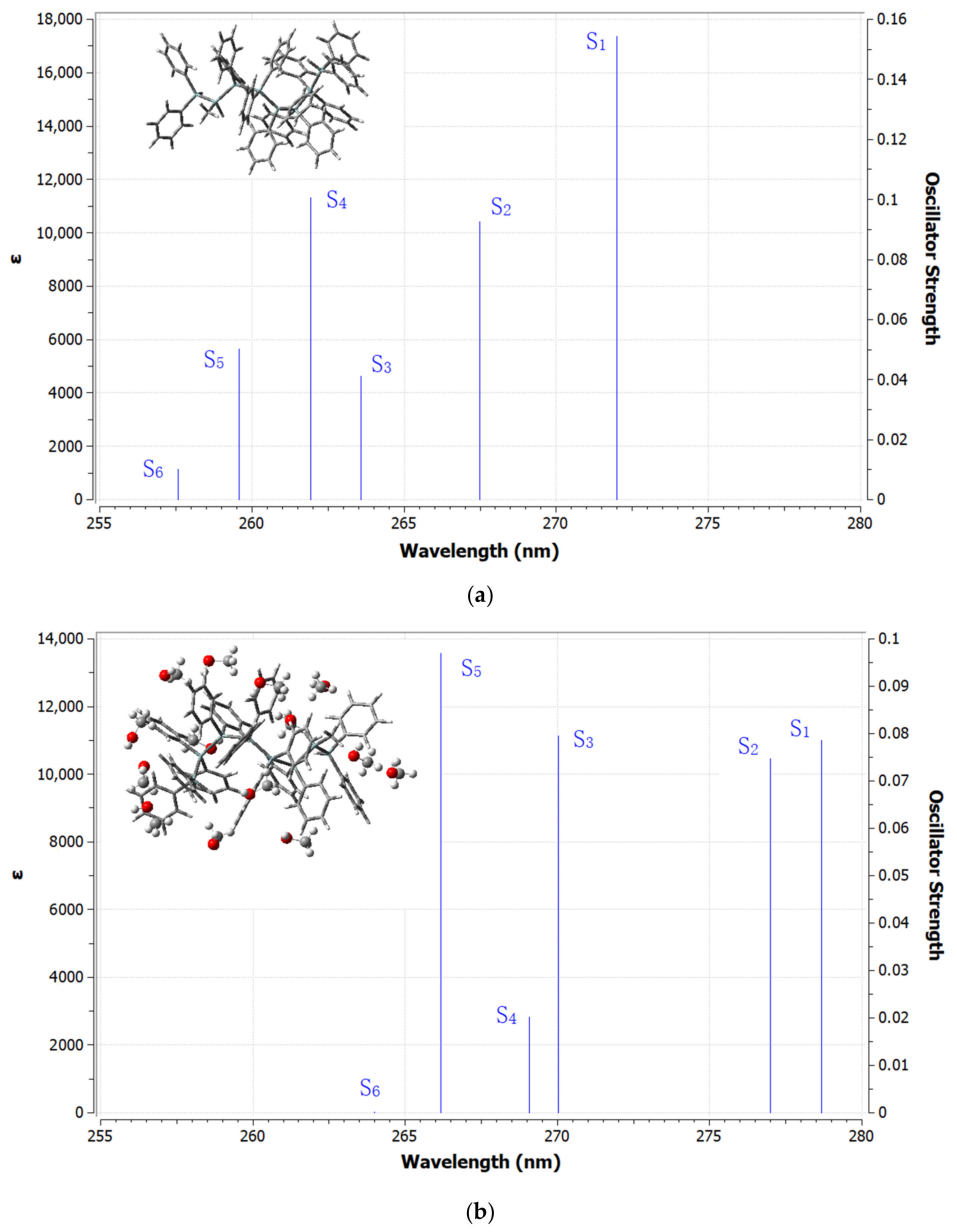
Theoretical electronic absorption spectra (UV-Vis): (**a**) theoretical UV-Vis spectrum of PSH oligomer (inset image: optimized PSH conformation); (**b**) theoretical UV-Vis spectrum of PSH oligomer under the influence of explicit methanol molecules (inset image: PSH conformer surrounded by 14 methanol molecules).

Figure 6 illustrates the theoretical electronic computed absorption spectra (UV-Vis) for PSH (Figure 6a), and for PSH in the presence of explicit methanol molecules (Figure 6b). Note that the calculations by the DFT method revealed that the moment dipole of the PSH oligomer changed from 1.23 Debye to 6.22 Debye when PSH was surrounded by 14 explicit methanol molecules.

It should be noticed that typically the first excited states in polysilanes are attributed to σ → σ* electronic transition owing to σ -electronic systems in the Si–Si backbone chain. In the experimental UV–Vis spectra of the polysilanes solution, these σ → σ* electronic transitions normally appear in the region of 300–400 nm, due to the long conjugated Si–Si chains [52]. In the theoretical UV-Vis spectra, the σ → σ* electronic transitions might appear below 300 nm due to the fact that much shorter Si–Si chains are subjected to the modeling procedure.

Thus, as reported in Figure 6a and Table 1, the first excited state S1 for the PSH oligomer was predicted at 270 nm, revealing an evident oscillator strength (f = 0.1543) and a light harvesting efficiency of LHE = 29.90%. As detailed by DFT theoretical computations, this electronic transition associated with the S1 excited state involved a mix of orbital configurations, where the main transition was attributed to HOMO → LUMO (80.57%) electronic configuration (Table 1).

For the second case, when PSH was surrounded by explicit methanol molecules (Figure 6b), the excited state S1 was red-shifted at 278.66 nm, disclosing an attenuated oscillator strength (f = 0.0785) and a reduced light harvesting efficiency of LHE = 16.54% (Table 2). Likewise, for this case, the first excited state S1 was associated with the main HOMO → LUMO (87.00%) electronic configuration (Table 2). Hence, the theoretical results, provided by TD-DFT method, suggested that the presence of methanol molecules in the vicinity of PSH can obviously decrease the oscillator strength associated to S1 excited state. These results are sustained by the experimental UV spectra (see Figure 5), showing that in the case of the polysilane (PSH) dispersion in methanol, the 350 nm UV absorption band is missing.

Therefore, the presence of this characteristic band in the UV absorption spectrum only in the case of PSCo dispersion in methanol should be related to the presence of the magnetic field generated by the Co ferrite core that interferes with the intramolecular electron transitions within the polysilane molecule and counteracts the attenuating effect of methanol.

**Figure 7:**
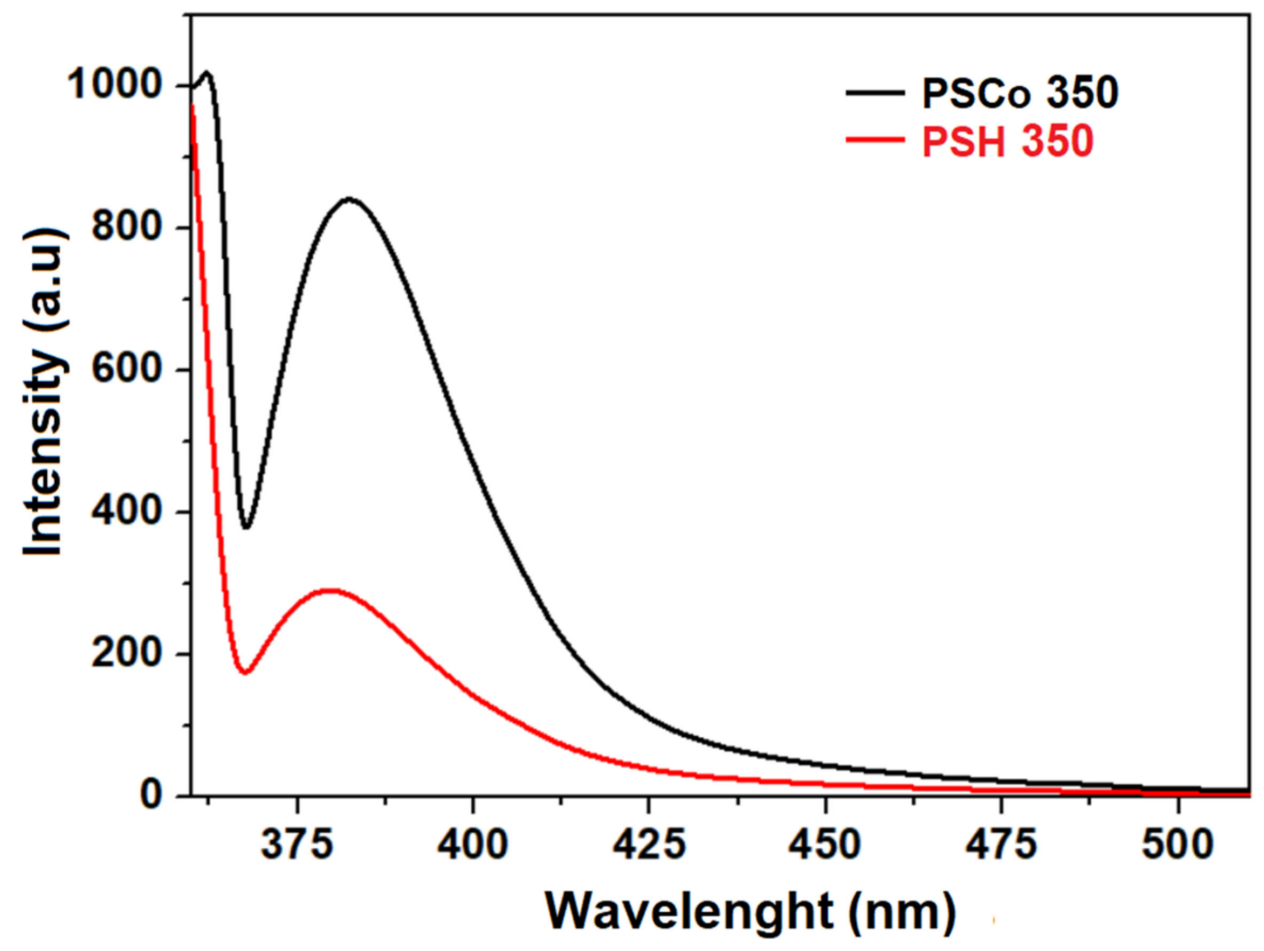
Comparison between the emission spectra of PSH and PSCo at excitation wavelength (λex) of 350 nm.

Further experiments were concerned with studying PSH and PSCo dispersions emission when the excitation wavelength was λex = 350 nm. Thus, it was observed that a strong amplification of the emission is present in the case of fluoromagnetic particles (Figure 7). Such an effect is usually explained by the so-called metal-enhanced fluorescence and occurs when the metal surface comes in contact with a fluorophore under light irradiation. This produces collective charge oscillation modes known as surface plasmons, at the surface of the metal particles. The resonant energy transfer from the surface plasmons to the fluorophore produces enhancement of the fluorescence. The effect was indeed observed in the case of noble metal nanoparticles such as Ag in contact with polysilane [53]. However, Co ferrite is very different from the pure Ag nanoparticles, and this explanation cannot be used for PSCo because ferrites do not generate surface plasmons that are matched with the polysilane emission wavelength.

Therefore, another possibility to explain the fluorescent enhancement should be related to the presence of the magnetic field. In general, the mechanism describing MFE for fluorescent polymers/MNP composites is still under debate. Usually, this magnetic field influence is easier to explain when the system is built with two molecular structures that play the role of charge donor and acceptor, respectively. In such cases, the mechanism concerning the magnetic field influence takes into account intermolecular excited states that have singlet and triplet configurations [54,55,56]. The problem becomes more challenging when MEF is observed for single molecular structures in contact with MNP, as in the case of PSCo particles. To explain MFE in such cases, it is important to clarify some important aspects. Basically, it is well-known that in a single molecule the photon absorption induces a HOMO to LUMO transition that can generate only singlet excitons according to the spin rule [57]. Further, by the so-called intersystem crossing (ISC), which is a spin-dependent process, the singlet excitons can be converted to triplets. This will redistribute the singlet and triplet populations within the excitonic states.

**Figure 8:**
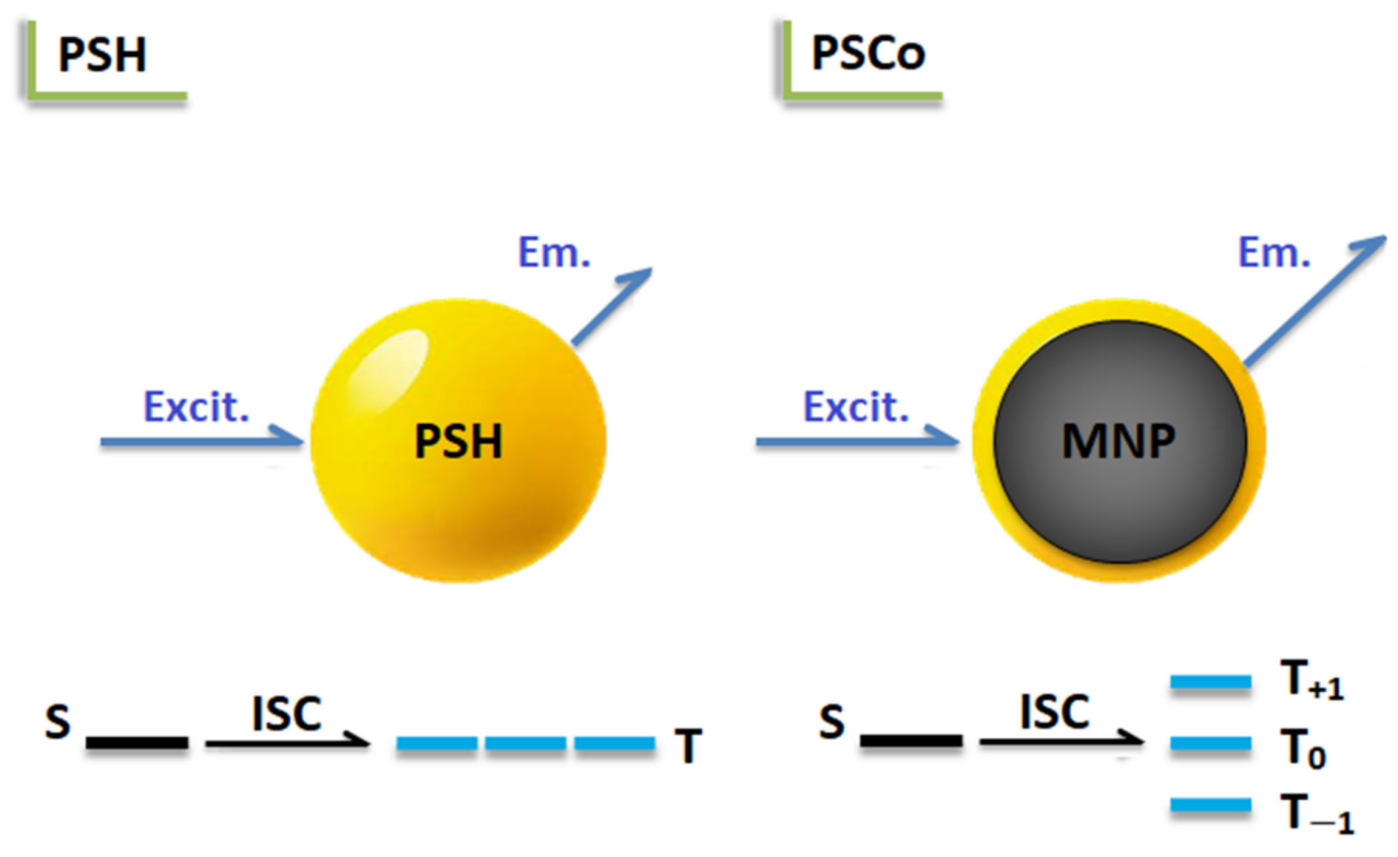
Schematic representation of the emission process in PSH describing the magnetic field effect on the excitonic states in PSCo (S—singlet state, ISC—intersystem crossing, and T—triplet state).

Studies concerning the behavior under light irradiation indicate that besides the excited singlet states, triplet states could result also in polysilanes by ISC processes [58,59]. When the PSH molecules are exposed to an external magnetic field such as in PSCo, the so-called Zeeman effect will induce splitting of the triplet level in sub-levels, as graphically represented in Figure 8.

This phenomenon will make improbable ISC from singlet to triplet excited states, and this action will qualitatively increase the population of singlet excitons. Because singlets are involved in the radiative relaxation of polysilane, MEF will produce, in this case, an enhancement of the fluorescence intensity.

## 3. Materials and Methods

### 3.1. Synthesis of Polysilane, Cobalt Ferrite, and Composite Particles

#### 3.1.1. Poly[diphenyl-co-methyl(H)]silane Synthesis (PSH)

**Scheme 2:**
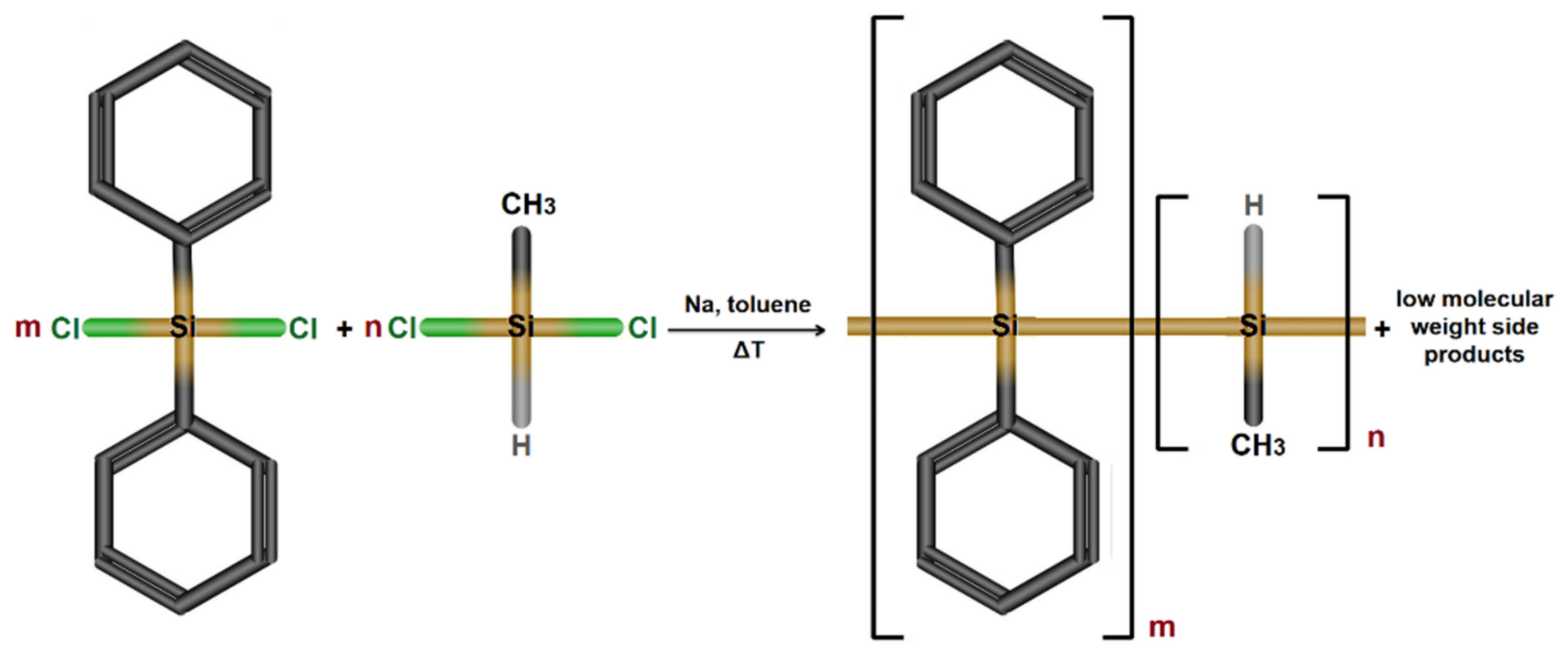
Schematic representation of the poly[diphenyl-co-methyl(H)]silane copolymer synthesis by using Wurtz coupling reaction (molar ratio between m/n ≈ 7/1).

Poly [diphenyl-co-methyl(H)]silane was obtained following the Wurtz reductive coupling method, using methyl(H)dichlorosilane and diphenyldichlorosilane as reagents. The preparation process has been described in detail in two previous papers [21,60]. The molar ratio C_6_H_5_Si/CH_3_(H)Si groups Mr = 7/1 ensure polysilane functionality, along with very good solubility in the usual organic solvents (toluene, THF, dichloromethane, etc.), as shown in Scheme 2. Additionally, methyl(H)silyl segments were introduced to provide high flexibility to the polysilane chain.

The polysilane copolymer has the following structural characteristics: 1H-NMR (400 MHz, CDCl_3_, δ): 0.48 (Si–CH_3_), 4.7 (Si–H), 7.28 (Si–C_6_H_5_). FTIR (KBr, cm^−1^): 3064 and 3045 (C–H_arom_), 2953 and 2893 (C–H_aliph_), 2100 (Si–H), 1424 and 1096 (Si–C_6_H_5_), 1243 (Si–CH_3_), 695 (Si–C), 461 (Si–Si). GPC (SLS-MALS, 1 mg/mL in THF): Mw = 4495 g/mol; Mw/Mn = 1.5.

#### 3.1.2. Synthesis of Cobalt Ferrite Particles (CoFe_2_O_4_)

The method used in this study to obtain the Co ferrite follows an experimental protocol presented previously [38,50]. In short, cobalt ferrite particles (CoFe_2_O_4_) were obtained by sol-gel autocombustion, using maleic acid as a chelating/combustion agent. In the first step, cobalt and iron nitrate solutions were mixed, in stoichiometric proportions. Then, a solution of maleic acid was added in the metal nitrates mixture in 1:1 molar ratio. Further, the as-obtained solution was heated at 80 °C on a water bath, under stirring, until a brown gel was formed. The gel was heated gradually up to 350 °C on a sand bath, until the self-ignition led to the formation of a dark powder. This powder was submitted to a thermal treatment in three stages: at 500 °C/5 h (pre-sinterization), at 700 °C/5 h, and at 900 °C/7 h (sinterization).

The full characterization of the obtained magnetic particles is provided in a previous work [50]. In brief, the stoichiometry of the cobalt ferrite was studied by means of Energy-Dispersive X-ray Fluorescence (ED-XRF), and the close agreement between experimental and theoretical chemical composition was proven. By means of X-ray diffraction analysis (XRD), the preparation of the pure cubic spinel crystal phase of cobalt ferrite, with no traces of detectable impurities, was demonstrated. The magnetic characterization demonstrated the ferrimagnetic behavior of the sample with a magnetization saturation value of 67.22 emu/g.

#### 3.1.3. Fluoromagnetic Particles Synthesis (PSCo)

The fluoromagnetic particles were prepared using an ultrasonic processor UP50H Hielscher equipped with a sonotrode (1 cycle at 100% amplitude). In the first step, 2 mL of 10% solution of PSH polysilane in THF solvent was prepared. Then, 100 mg of ferrite were added to the PSH solution and the mixture was subjected to ultrasound for 60 s. The obtained suspension was immediately poured, in drops, over 50 mL of methanol, under stirring, obtaining a white-gray precipitate. The purification was done by washing-decanting from methanol solution under an external magnetic field. After three cycles of separation-washing, the solid material was filtered and dried in a vacuum oven at 60 °C (until constant weight). Finally, approximately 70 mg of the final PSCo dry product as a white-gray powder was obtained. The amount of CoFe_2_O_4_ was found to be 8.33% (*w*/*w*) for the composite material.

### 3.2. General Methods of Characterization

High-resolution transmission electron microscopy (HR-TEM) was conducted on a HITACHI HT7700 microscope (Hitachi High Technologies Corporation, Tokyo, Japan) operated at 120 kV in high-contrast mode. The instrument was equipped with a Bruker XFlash 6 energy-dispersive X-ray (EDX) detector. The samples were prepared by dispersing approximately 0.1 g of the solid material (polymer, ferrite, or fluoromagnetic powder) in 10 mL methanol using an ultrasonication bath. Further, small droplets of these dispersions have been placed on copper grids (300 mesh) coated with an amorphous carbon film (Ted Pella, Redding, CA, USA) and dried at 50 °C in vacuum for 24 h prior to the measurements.

Fourier transform-infrared (FTIR) spectra were registered on a Bruker Vertex 70 spectrometer (Ettlingen, Germany) in transmittance mode in the range 4000–600 cm^−1^ by accumulation of 32 scans, at a resolution of 2 cm^−1^. The samples in the form of powder were deposited on KBr pellets.

The UV–Vis absorption and emission spectra of the polymeric matrix and fluoromagnetic particles were obtained using samples in the form of dispersions. These have been prepared by dispersing 20 mg of material in 10 mL methanol. Then, the dispersions were allowed to rest for 30 min, in order to form two layers. The clear layer was separated and used further. The absorbance measurements at room temperature indicated that these diluted dispersions were stable within 45–50 min before a noticeable decay of the maximum. However, in order to have good reproducibility, the dispersions were maintained under stirring in the ultrasonication bath for 30 s before each measurement. The electronic absorption spectra were recorded on an Analytik Jena (Jena, Germany) SPECORD UV/Vis 210 (plus) spectrophotometer using a pair of quartz cells of 10 mm optical length.

Fluorescence spectra were acquired using a Perkin Elmer LS55 (PerkinElmer, Inc., Waltham, MA, USA) luminescence spectrometer in 10 mm quartz cells. The excitation wavelength was selected according to the UV measurements at 350 nm. The excitation and emission slits were set at 15 and 3 nm, respectively, for all the measurements.

Magnetic measurements were performed using a vibrating sample magnetometer LakeShore 8607 VSM system. Before the experiment, the sample was demagnetized in an alternating field.

### 3.3. Computational Protocol

The computational chemistry approach was done at the level of PM6 semi-empirical and density functional theory (DFT) by using the Gaussian16 program [51] on a Dell Precision Workstation T7910 (2 × Intel Xeon OCTA Core E5–2630). The theoretical results of molecular modeling were displayed and analyzed by means of the GaussView 6 program [61]. The geometry optimization of the studied structure of oligomeric polysilane (PSH) was performed in the gas phase at the level of the semi-empirical PM6 method. Afterward, on this optimized conformation, the single-point energy calculations were performed using a more advanced method, i.e., DFT/B3LYP/6-31G**. Likewise, the theoretical electronic absorption spectra were calculated at the level of the time-dependent density functional theory (TD-DFT) using the same method and basis set, i.e., TD-DFT/B3LYP/6-31G**. For solvating the polysilane oligomer with explicit methanol molecules, we employed the YASARA-Structure software package [62].

## 4. Conclusions

Precipitation of soluble poly[diphenyl-co-methyl(H)]silane in a Co ferrite suspension in methanol under ultrasound exposure generates fluoromagnetic particles. The resulting spherical particles of about 500 nm diameters contain a magnetic core made of about 10 nm ferrite particles wrapped in a 40 nm polysilane layer. These structures preserve both the magnetic and fluorescent properties of the components. In addition, the magnetic core has a strong influence on the electron transitions induced by light irradiation of the σ-conjugated polysilane. The fluorescence enhancement effect due to the internal magnetic field exposure is noteworthy. This is a rare phenomenon for polymers and is the first example describing it for polysilanes. Since the preparation method of this composite material is very facile, such fluoromagnetic particles could be used in detection systems based on the fluorescence response.

## Figures and Tables

**Table 1:** Summary * of theoretical electronic-absorption spectra characteristics calculated by TD-DFT method (at B3LYP/6-31G** level of theory) for the oligomer PSH.

Excited State	Λ (nm)	E (eV)	f	LHE%	Main Transition Assignments (Contribution, %)
S0 → S1	272.00	4.5582	0.1543	29.90%	HOMO → LUMO (80.57%)
					HOMO-1 → LUMO (10.66%)
					HOMO → LUMO + 1 (2.49%)
S0 → S2	267.48	4.6353	0.0925	19.18%	HOMO → LUMO + 1 (69.43%)
					HOMO-1 → LUMO (12.48%)
					HOMO → LUMO (7.28%)
S0 → S3	263.58	4.7038	0.0410	9.01%	HOMO-1 → LUMO (51.83%)
					HOMO-1 → LUMO + 1 (16.80%)
					HOMO → LUMO + 1 (15.19%)
S0 → S4	261.93	4.7335	0.1005	20.66%	HOMO-1 → LUMO + 1 (41.77%)
					HOMO → LUMO + 2 (25.65%)
					HOMO-1 → LUMO (9.25%)
S0 → S5	259.57	4.7765	0.0502	10.92%	HOMO → LUMO + 2 (52.06%)
					HOMO-1 → LUMO + 1 (17.84%)
					HOMO-1 → LUMO + 3 (3.33%)
S0 → S6	257.59	4.8133	0.0102	2.32%	HOMO-2 → LUMO (42.84%)
					HOMO-1 → LUMO + 2 (15.98%)
					HOMO → LUMO + 3 (7.80%)

* where λ (nm)-absorption wavelength (calculated vertical transition); E (eV)-excitation energy; f-oscillator strength; and LHE%-Light harvesting efficiency (%): LHE% = (1 − 10^−f^ ) × 100 (%).

**Table 2:** Summary * of theoretical electronic-absorption spectra characteristics calculated by TD-DFT method (at B3LYP/6-31G** level of theory) for the oligomer PSH surrounded by 14 methanol (CH_3_OH) molecules.

Excited State	Λ (nm)	E (eV)	f	LHE%	Main Transition Assignments (Contribution, %)
S0 → S1	278.66	4.4493	0.0785	16.54%	HOMO → LUMO (87.00%)
					HOMO → LUMO (3.64)
S0 → S2	277.00	4.4759	0.0747	15.80%	HOMO-1 → LUMO (76.48%)
					HOMO → LUMO + 1 (10.76%)
					HOMO → LUMO + 2 (2.77%)
S0 → S3	270.03	4.5915	0.0795	16.73%	HOMO → LUMO + 1 (75.31%)
					HOMO-1 → LUMO (12.61%)
					HOMO-1 → LUMO + 3 (2.63%)
S0 → S4	269.09	4.6075	0.0202	4.54%	HOMO-1 → LUMO + 1 (74.19%)
					HOMO → LUMO + 3 (7.30%)
					HOMO → LUMO + 2 (4.26%)
S0 → S5	266.18	4.6580	0.0970	20.02%	HOMO → LUMO + 2 (67.57%)
					HOMO-1 → LUMO + 2 (15.83%)
					HOMO-1 → LUMO (2.30%)
S0 → S6	263.99	4.6966	0.0002	0.05%	HOMO-1 → LUMO + 2 (64.07%)
					HOMO → LUMO + 2 (12.91%)
					HOMO → LUMO + 3 (2.79%)

* where λ (nm)-absorption wavelength (calculated vertical transition); E (eV)-excitation energy; f-oscillator strength; and LHE%-Light harvesting efficiency (%): LHE% = (1 − 10^−f^ ) × 100 (%).

## Data Availability

Not applicable.

## Sample Availability

Samples of the compounds CoFe_2_O_4_, PSH, and PSCo are available from the authors.
